# Climate Warming and Tick-borne Encephalitis, Slovakia

**DOI:** 10.3201/eid1603.081364

**Published:** 2010-03

**Authors:** Martin Lukan, Eva Bullova, Branislav Petko

**Affiliations:** University of Žilina, Tatranská Javorina, Slovakia (M. Lukan); Slovak Academy of Sciences, Košice, Slovakia (M. Lukan, E. Bullova, B. Petko); Catholic University Ružomberok Faculty of Health, Ružomberok, Slovakia (B. Petko)

**Keywords:** Tick-borne encephalitis, climate warming, central Europe, Slovakia, vector-borne infections, viruses, dispatch

## Abstract

Increased tick-borne encephalitis (TBE) cases have been reported in central Europe. To investigate temporal trends in the altitude at which TBE cases occur in Slovakia, we analyzed the number of TBE cases during 1961–2004. Since 1980, TBE cases moved from lowlands to submountainous areas, most likely because of rising temperature.

The recent increase in incidence of tick-borne encephalitis (TBE) in central and eastern Europe, especially since 1990, has been attributed to climate warming (*1*–*5*) or various socioeconomic factors (*6*,*7*). Climate warming in Europe during the past decades has been shown to influence the distribution of *Ixodes ricinus* ticks, the main TBE vector, in several European countries (*4*,*5*,*8*). In central Europe, a sharp increase of TBE has been reported (*9*,*10*). Zeman and Beneš showed that global warming affected the geographic and temporal distribution of TBE cases in the Czech Republic (*2*). Similar development of TBE vertical distribution could be expected in neighboring Slovakia. To investigate temporal trends in the altitude at which TBE cases occur (altitude for TBE) in Slovakia and TBE response to climate warming, we analyzed the total number of TBE cases recorded for persons in Slovakia during 1961–2004.

## The Study

Since the 1952 outbreak of TBE in Rožňava, Slovakia, all registered cases of TBE have been required to be reported to the National Health Institute. We analyzed 1,786 TBE cases registered in Slovakia by the Regional Institute of Health during 1961–2004.

Location where infection occurred was tracked to the level of cadastral unit. We calculated the average altitude of cadastral units corresponding to the reported TBE cases by using an altitudinal model of the country and ArcGIS 9.2 software (www.ESRI.com). A TBE focus was defined as a location at which TBE infection occurred at least 1 time in a given year. The yearly average altitude for TBE was plotted against time, and temporal trends were identified by linear regression analysis. Frequency distribution of TBE foci in relation to altitude was plotted, and 5-year periods were aggregated. To eliminate locations with single, possibly accidental, cases of the disease, we considered established TBE foci where TBE had occurred in at least 2 of 5 years .The series of yearly mean altitudes of TBE foci was tested against the null hypothesis of random elevation by using the Spearman rank correlation (2-tailed test; null hypothesis = temporal and altitudinal rankings are uncorrelated) and the test for stationarity of Kwiatkowski et al. (*11*) (null hypothesis = time series in question is stationary; i.e., no change over time). A series of yearly mean altitudes for TBE was analyzed for correlations with mean yearly temperature and precipitation derived from 12 meteorologic stations throughout Slovakia. Climate data were kindly provided by the Slovak Hydrometeorological Institute (www.shmu.sk). Statistical tests were performed by using SPSS 14.0 for Windows (Chicago, IL, USA) and Gretl 1.8.5 (*12*).

**Figure 1:**
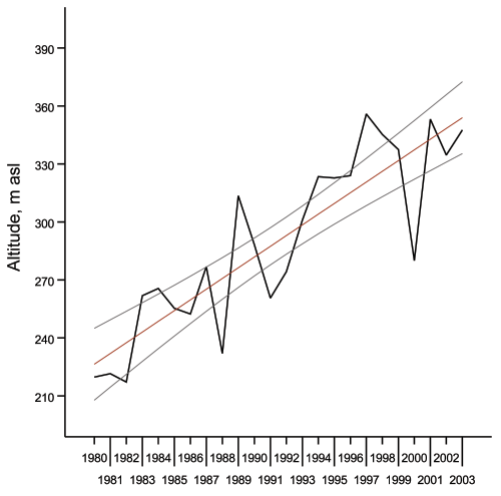
Mean altitude of reported cases of tick-borne encephalitis (TBE), Slovakia, 1980–2004. Black line, mean altitude; red line, linear least-square fit; gray lines, 95% confidence intervals. asl, above sea level.

During 1961–1979, the mean altitude for TBE varied between 180 m and 340 m above sea level. Time series of mean altitudes for TBE showed random elevation, and statistical analysis showed no temporal trend. During this period, no temporal trend in the average annual air temperature was noted. However, during the following period, 1980–2004, the mean altitudes for TBE showed nonrandom variation over time. The gradual increase is shown in Table 1. An analysis of trends (linear least-square fit) showed a mean ± SD annual ascension rate of 5.32 ± 0.63 m; R^2^ = 0.76, p<0.001 (Figure 1). The relationship can be expressed as the following equation: Annual rise in the mean altitude of TBE incidents (meters above sea level) = (222.80 + 5.32) × (year from 1980 inclusive) ± 0.63.

**Table 1:** Nonparametric test and test of stationarity for mean altitude and mean annual air temperature with regard to TBE, Slovakia, 1980–2004*

Data	Test values				
	R_S_	p value†		KPSS	p value†
Mean annual air temperature	0.55	0.01		0.56	<0.05
Mean TBE altitude	0.87	<0.001		0.86	<0.01

*TBE, tick-borne encephalitis; Rs, Spearman rank correlation test, a nonparametric test; KPSS, Kwiatkowski-Phillips-Schmidt-Shin test, a test of stationarity (no change with time). †Probability of adopting the null hypothesis of randomness (Spearman R_S_) and stationarity (KPSS).

The observed rise in mean altitude for TBE corresponds with a mean ± SD rate of TBE ceiling (uppermost limit) rise of ≈5.4 ± 1.7 m yearly during the past 3 decades in the neighboring Czech Republic (*2*). During the same frame, the mean annual temperature showed a gradual rise (Table 1). An analysis of trends (linear least-square fit) showed an annual increase of 0.067°C ± 0.019°C (R^2^ = 0.36, p = 0.002).

**Figure 2:**
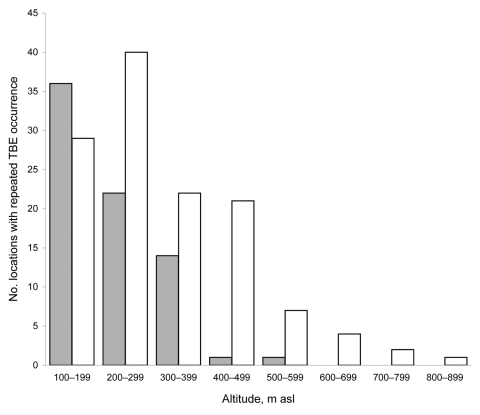
Comparison between altitudinal distribution of tick-borne encephalitis (TBE) foci during 2 time periods, 1980–1984 (gray bars) and 2000–2004 (white bars), Slovakia. asl, above sea level.

The mean altitude for TBE in this period was significantly correlated with mean annual air temperature (Table 2). No significant correlation between the mean altitude for TBE and precipitation could be found. The closest correlation was detected between the mean altitude for TBE and mean annual air temperature of the 3 preceding years. This correlation indicates that the mean altitude for TBE positively responds to climate warming, with a lag of several years. A similar phenomenon was described by Zeman and Beneš (*2*). At the beginning of the observed period of change, 1980–1984, 48.6% of TBE foci were found at <200 m (Figure 2), 21.6% were found at >300 m, and the highest with repeated reports of TBE was 550 m. During 2000–2004 only 23.0% of locations with repeated reports of TBE were found at <200 m, 27.8% of all locations were found at >400 m, and 5.6% of all TBE foci were found at >600 m (Figure 2). During this period, the highest location with TBE occurrence repeated for several years was 832 m. The total number of lowland TBE foci at <200 m decreased from 36 during 1980–1984 to 29 during 2000–2004.

**Table 2:** Relationship between mean annual air temperature and mean altitude of tick-borne encephalitis cases, Slovakia, 1980–2004*

Temperature lag, y	Correlation coefficient	p value
Mean (1–3)	0.689†	0.000
0	0.30‡	0.032
–1	0.466‡	0.019
–2	0.433‡	0.031
–3	0.438‡	0.028

*Nonparametric testing (Spearman rank correlation test). †Correlation is significant at p<0.01 (2-tailed). ‡Correlation is significant at p<0.05 (2-tailed).

In contrast, the total number of TBE foci at >400 m was only 2 during 1980–1984 and increased to 35 during 2000–2004. The altitudinal distribution of TBE foci during 1980–1984 differed significantly from that during 2000–2004 (log-likelihood ratio 31.302, df = 7, p<0.001). The number of lowland TBE foci became significantly lower than in the beginning, a finding that corresponds with the predictions of Randolph and Rogers about the gradual disappearance of TBE from the lowlands of central Europe (*7*). The dramatic rise in the number of TBE foci at >400 m between 1980–1984 (2 foci) and 2000–2004 (35 foci) is too great to be explained by only socioeconomic factors, such as particular changes in land use, which could increase the range of habitats suitable for tick survival at higher altitudes.

## Conclusions

If the observed trend continues, the number of TBE foci in the mountain areas >500 m will probably increase in future decades. Whether this would affect the total number of TBE cases is a matter for discussion. Higher areas are less densely inhabited by local residents but often visited for leisure activities and recreation. The possibility of TBE emergence should be therefore considered by the management of recreation facilities and tourist resorts in areas with habitats suitable for TBE vectors.
